# Advanced Endoscopic Imaging and Interventions in GERD: An Update and Future Directions

**DOI:** 10.3389/fmed.2021.728696

**Published:** 2021-11-29

**Authors:** Rupinder Mann, Mahesh Gajendran, Abhilash Perisetti, Hemant Goyal, Shreyas Saligram, Chandraprakash Umapathy

**Affiliations:** ^1^Department of Internal Medicine, Saint Agnes Medical Center, Fresno, CA, United States; ^2^Paul L. Foster School of Medicine, Texas Tech University Health Sciences Center, El Paso, TX, United States; ^3^Department of Gastroenterology and Hepatology, The University of Arkansas for Medical Sciences, Little Rock, AR, United States; ^4^Department of Gastroenterology and Advanced Endoscopy, Parkview Health, Fort Wayne, IN, United States; ^5^The Wright Center for Graduate Medical Education, Scranton, PA, United States; ^6^Division of Gastroenterology, Long School of Medicine, University of Texas Health San Antonio, San Antonio, TX, United States

**Keywords:** gastroesophageal reflux disease (GERD), endoscopic reflux therapy, narrow band imaging (NBI), endoscopy, Barrett's esophagus (BE)

## Abstract

Gastroesophageal reflux disease (GERD) is one of the most common gastrointestinal diseases encountered in primary care and gastroenterology clinics. Most cases of GERD can be diagnosed based on clinical presentation and risk factors; however, some patients present with atypical symptoms, which can make diagnosis difficult. An esophagogastroduodenoscopy can be used to assist in diagnosis of GERD, though only half of these patients have visible endoscopic findings on standard white light endoscopy. This led to the development of new advanced endoscopic techniques that enhanced the diagnosis of GERD and related complications like squamous cell dysplasia, Barrett's esophagus, and early esophageal adenocarcinoma. This is conducted by improved detection of subtle irregularities in the mucosa and vascular structures through optical biopsies in real-time. Management of GERD includes lifestyle modifications, pharmacological therapy, endoscopic and surgical intervention. Minimally invasive endoscopic intervention can be an option in selected patients with small hiatal hernia and without complications of GERD. These endoscopic interventions include endoscopic fundoplication, endoscopic mucosal resection techniques, ablative techniques, creating mechanical barriers, and suturing and stapling devices. As these new advanced endoscopic techniques are emerging, data surrounding the indications, advantages and disadvantages of these techniques need a thorough understanding.

## Introduction

Gastroesophageal reflux disease (GERD) is one of the most common gastrointestinal diseases of the western world, with increasing morbidity (1, 2). The estimated prevalence of GERD worldwide is 8–33% (3). A systematic review showed the estimated prevalence of GERD to be 18.1–27.8% in North America, 8.8–25.9% in Europe, and 2.5–7.8% in East Asia (1). Due to the common use of over-the-counter medications for GERD, the true incidence of the disease is likely underestimated (4). GERD is known to involve all races, age groups, and all genders (1, 3). Genetic and environmental risk factors like obesity, smoking, *Helicobacter pylori* infection, hiatal hernia, pregnancy, medications, and food are associated with this disease (5–10). A meta-analysis showed higher prevalence in smokers [Odds Ratio (OR) 1.26; 95% Confidence Interval (CI) 1.04–1.52], obese individuals (OR 1.73; 95% CI 1.46–2.06), age ≥ 50 years (OR 1.32; 95% CI 1.12–1.54), and women (OR 1.12; 95% Cl 1.05–1.21) (11). GERD is diagnosed in routine clinical practice based on typical clinical symptoms and treated empirically with a proton pump inhibitor (PPI) trial unless a patient has alarming symptoms, which include dysphagia, anemia, weight loss, hematemesis, and odynophagia (12–14). The patient who does not respond to the empiric PPI trial or those with alarming symptoms should undergo an esophagogastroduodenoscopy (EGD) to evaluate for complications like Barrett's esophagus, esophagitis, peptic ulcer disease, or esophageal cancer (3). Some of the complications, like squamous cell dysplasia, Barrett's esophagus with dysplasia, and early adenocarcinoma, can be missed with regular EGD due to subtle changes in the mucosa (15, 16). Advanced diagnostic endoscopic techniques like high-resolution, high-magnification endoscopy, confocal laser endomicroscopy, wireless capsule endoscopy, autofluorescence imaging, narrow-band imaging, and chromoendoscopy have been developed to improve the accuracy of the endoscopic diagnosis.

**Figure 1:**
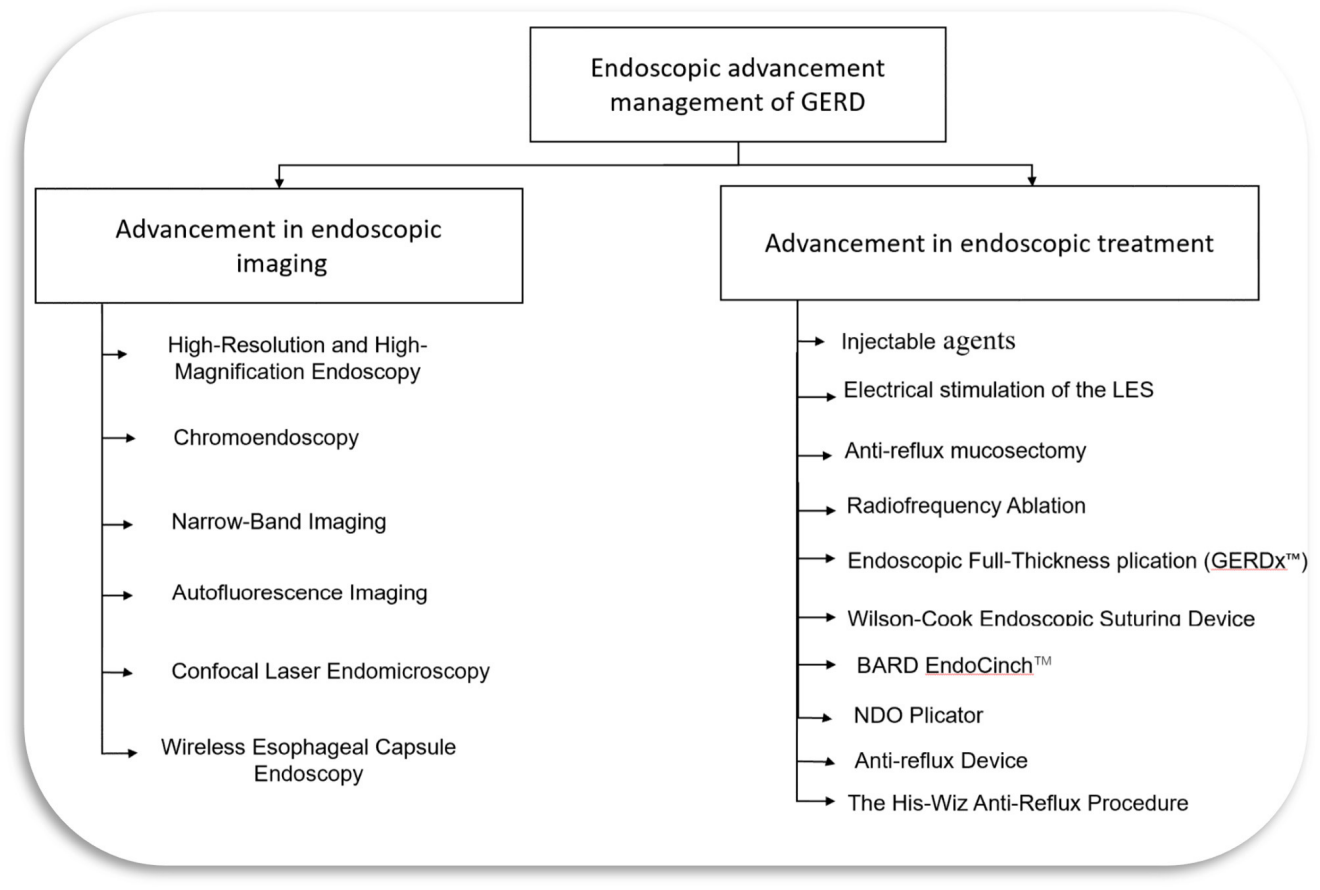
Endoscopic management of GERD. LES, lower esophageal sphincter; GERD, Gastroesophageal reflux disease.

Although medical management with PPI and lifestyle modifications is considered standard therapy for GERD, around 20–30% of patients with erosive and 40% with non-erosive reflux disease do not respond to PPIs (14, 17). Patients who do not respond to PPI or refuse to take long-term medical therapy due to potential side effects can be a candidate for surgical or endoscopic intervention for treatment (4, 18). Endoscopic treatment options include endoscopic anti-reflux techniques utilizing injection devices, suturing, plicating or stapling devices, and radiofrequency ablation (4, 19). This review will discuss various advanced endoscopic diagnostic techniques and minimally invasive endoscopic treatment modalities for GERD (Figure 1).

## Advances in Endoscopic Imaging for Gerd

Conventional EGD allows visualization of mucosal breaks and to obtain biopsies to confirm the diagnosis of erosive GERD. There are no mucosal breaks on conventional EGD in non-erosive reflux disease (NERD), but these patients have reflux symptoms. Similarly, biopsies from columnar mucosa in Barrett's esophagus reveal metaplasia only in 40–60% of cases since the metaplastic tissue is patchy (20). Advanced endoscopic imaging techniques have been shown to improve the diagnosis of GERD. These techniques are described below (Table 1).

**Table 1:** Endoscopic imaging for GERD diagnosis.

**Endoscopic imaging test**	**Study**	**Number of patients**	**Results**
Magnification Endoscopy	Retrospective observational study (23)	500 procedures were included	Use of dual focus magnification and high-definition endoscopy associated with odd ratio of 1.87 (95% Cl: 1.11–3.12) for detection of pathology on EGD
Chromoendoscopy	Meta-analysis and systematic review (29)	843 patients	Diagnostic yield for detection of dysplasia or cancer in patients with BE increases by 34% (95% Cl: 20–56%; 0 < 0.0001)
Narrow-band imaging (NBI)	Meta-analysis and systematic review (35)	502 patients	Sensitivity and specificity of NBI is 0.91 (95% CI: 0.86–0.94) and 0.85 (95% Cl: 0.76–0.92) on a per-patient for specialized intestinal metaplasia, whereas for high-grade dysplasia, respectively, sensitivity and specificity are 0.91 (95% Cl: 0.75–0.98) and 0.64 995% Cl: 0.59–0.68).
Autofluorescence imaging (AFE)	Multicenter randomized controlled trial (40)	130 patients	On per patient basis, AFE and conventional endoscopy diagnostic yield was 12 and 5.3%, respectively.
Confocal laser endomicroscopy (CLE)	Meta-analysis (43)	789 patients	Per patient analysis showed pooled sensitivity and specificity of 89% (95% Cl: 0.82–0.94) and 83% (95% Cl: 0.87–0.90) respectively, for detection of neoplasia in BE.
Wireless esophageal capsule endoscopy	Meta-analysis (46)	618 patients	Pooled sensitivity and specificity for diagnosis of BE is 77 and 86%, respectively.

*BE, Barrett's esophagus*.

### High-Resolution and High-Magnification Endoscopy

Magnification enlarges the images, and high resolution improves the ability to detect minute details. Advances in optical engineering have made it possible to have a movable zoom lens in the tip of the magnification endoscopes that can provide up to 150-fold magnification and high-resolution endoscopes that use 850,000 pixels to provide high-resolution images (21). In a comparative study, consecutive patients who presented for EGD were divided into those with reflux symptoms (NERD group, *N* = 39) and non-reflux patients (control group, *N* = 39) with the help of a questionnaire; the endoscopists were blinded to the presence of reflux symptoms. On examination with magnification endoscope, a higher percentage of patients in the NERD group showed endoscopic changes of minimal change esophagitis when compared to the control group (64.10 vs. 20.5%, *P* = 0.003). The combination of endoscopic changes and one of the histologic abnormalities (basal cell hyperplasia or elongation of papilla) were found to have sensitivity, specificity, positive predictive value (PPV), and negative predictive value (NPV) of 62, 74, 67, and 67%, respectively, for NERD prediction. After 4 weeks of treatment with esomeprazole, no significant difference was seen in the endoscopic and histologic characteristics between the NERD and the control group (22). In another retrospective study, 500 procedures for patients coming for direct-to-test upper gastrointestinal endoscopy were included. Out of 500, 94 procedures were performed using dual-focus magnification high-definition endoscopy, and it was associated with 87% increased odds (OR 1.87, 95% Cl 1.11–3.12) for detecting significant mucosal pathology (23). High-resolution and magnification endoscopy can improve the detection of abnormal mucosal changes both endoscopically and histologically, especially in case of minimal change esophageal disease.

### Chromoendoscopy

Chromoendoscopy was introduced in the 1970s to improve the localization of abnormal mucosa in the esophagus and characterize such mucosa (24). In chromoendoscopy, a contrast agent is used to stain tissue during gastrointestinal endoscopy to improve different mucosa characterization. Currently, two groups of dyes are being used for chromoendoscopy. The first group, called vital stained dye, includes Lugol's solution, methylene blue, Congo red Lugol's solution, and toluidine blue. These dyes are rapidly absorbed by the normal squamous epithelial cells. The second group is called non-vital dye, and it includes indigo carmine and crystal violet. These dyes are not absorbed into cells but highlight the mucosal patterns in different structures by filling mucosal pits and folds. Chromoendoscopy is often used along with high-resolution and high-magnification endoscopy (21, 25, 26). Yoshikawa et al. conducted a study to determine the usefulness of Lugol chromoendoscopy for the diagnosis of NERD. Four of 42 individuals (9.5%) in the control group and 22 of 61 patients (36.1%) in the typical reflux symptoms group had visible esophagitis seen on conventional white light EGD. The remaining 38 patients in the endoscopy negative asymptomatic control group and 39 patients in the NERD group underwent Lugol chromoendoscopy. Out of 38, one individual in the control group and 19/39 in the NERD group had unstained streaks observed in the distal esophagus (*p* < 0.0001). The unstained streaks by Lugol chromoendoscopy are indicative of mucosal injury, which was not detectable by conventional endoscopy. The histological examination of biopsied unstained mucosa showed more typical pathologic changes, significantly thicker basal cell layer (30.9 vs. 12.3% of total epithelial thickness, *p* < 0.01), longer papillae (57.9 vs. 38.1% of total epithelial thickness, *p* < 0.01) and higher numbers of intraepithelial lymphocytes (9.6 vs. 6.0 per 3 high-power fields, *p* < 0.01) when compared with stained mucosa (27).

In another comparative study, 155 patients (62 with typical reflux symptoms and 93 without esophageal symptoms) were enrolled for virtual chromoendoscopy called Fuji Intelligent Color Enhanced (FICE) to evaluate if it will improve the diagnosis of minimal lesions on endoscopy and symptoms associated with a minimal lesion in patients with NERD. Among 155 patients, 113 had normal endoscopy of the esophagus, and forty-two had minimal endoscopic lesions on conventional endoscopic examination. Among 113 patients with normal findings on conventional endoscopy, 104 had normal mucosa, and nine had minimal endoscopic lesions on FICE. In comparison, all forty-two patients had minimal endoscopic lesions both on conventional endoscopy and FICE. Males were found to have a higher diagnosis of minimal endoscopic lesions than females (OR 4.1, *p* < 0.001, 95% CI: 1.9–8.9 for conventional endoscopy and OR 4.2, *p* < 0.001, 95% CI: 1.9–9.0 for FICE). There was no association between diagnosis of minimal endoscopic lesion and age, use of NSAIDS, PPIs, smoking, alcoholism, and reflux symptoms. Although there was an improvement in the minimal endoscopic lesion diagnosis with FICE, it is observer-dependent for conventional endoscopy and FICE (28) (Supplementary Figure S2). In a meta-analysis of 14 studies with 843 patients, advanced imaging techniques (chromoendoscopy and virtual chromoendoscopy) increased diagnostic yield for detection of dysplasia or cancer in patients with Barrett's esophagus by 34% (95% Cl: 20–56%, *p* < 0.0001). Furthermore, there was no difference between chromoendoscopy and virtual chromoendoscopy (*p* = 0.45) (29).

### Narrow-Band Imaging

NBI is a technique that utilizes a spectral narrow-band filter for object illumination and to detect mucosal pattern changes due to histological changes (13, 25) (Supplementary Figure S1). NBI helps the examination of mucosa without the need for chromoendoscopy as spectral narrow-band filters help with imaging of the mucosa and vascular patterns of the esophagus (26, 30). It also enhances the contrast between esophageal mucosa and gastric mucosa, as hemoglobin is the main chromophore in esophageal tissue in the visible wavelength range, which is in the wavelength range for NBI (26, 31). It can be combined with high-resolution and high-magnification endoscopy. It enables highlighting patterns of “intrapapillary capillary loops,” which contains abnormal figures indicating inflammatory process and cancer when used along with magnification endoscopy (32, 33). An international prospective randomized controlled trial (RCT) enrolled 123 patients with Barrett's esophagus randomized to high-definition white-light endoscopy or NBI followed by other procedures in 2–8 weeks to compare detection of intestinal metaplasia and neoplasia in Barrett's esophagus by these two procedures. During high-definition white-light endoscopy, biopsies were taken as per the Seattle protocol, and only target biopsies were taken during NBI examination based on mucosal and vascular patterns. Both NBI and high-definition white-light endoscopy were equally effective in detecting intestinal metaplasia (92%). However, for the detection of areas with dysplasia, NBI performed better than high-definition white-light endoscopy (30 vs. 21%, *p* = 0.01), and it required fewer biopsies per patient (3.6 vs. 7.6, *p* < 0.0001) (34).

A meta-analysis of 11 studies showed that NBI has sensitivity and specificity of 0.91 (95% CI: 0.86–0.94) and 0.85 (95% CI: 0.76–0.92) on a per-patient, and 0.97 (95% CI: 0.95–0.98) and 0.64 (95% CI: 0.59–0.68) on a per-lesion basis for specialized intestinal metaplasia diagnosis in the Barrett's esophagus, respectively. Similarly, NBI has sensitivity and specificity of 0.91 (95% CI: 0.75–0.98) and 0.95 (95% Cl: 0.91–0.97) on a per-patient, and 0.69 (95% CI: 0.63–0.74) and 0.90 (95% CI: 0.88–0.91) on a per-lesion basis for high-grade dysplasia in the Barrett's esophagus, respectively (35). NBI improves the diagnosis of GERD, so it can be used as an adjunct along with conventional endoscopy.

### Autofluorescence Imaging

Autofluorescence imaging (AFI) is based on the principle that there is an emission of light with a longer wavelength on the excitation of tissues with the light of a shorter wavelength. There are some endogenous tissue molecules in our gastrointestinal tract, such as flavins, collagen, nicotinamide adenine dinucleotide phosphate, that are fluorophores and emit fluorescence light with a longer wavelength when excited with short-wavelength light (26, 36–39). Dysplastic and non-dysplastic Barrett's esophagus resulted in different autofluorescence characteristics due to different fluorophore contents (36, 39). In a multicenter RCT, 130 patients with Barrett's esophagus were randomly assigned to either Autofluorescence endoscopy (AFE)-target biopsy plus four-quadrant biopsies or conventional endoscopic surveillance with four-quadrant biopsies. After a mean of 10 weeks, these patients were re-examined with the alternative method. AFE diagnostic yield for adenocarcinoma/high-grade dysplasia was 12% compared to 5.3% for conventional endoscopy on a per-patient basis. However, AFE sensitivity was only 42% for detecting adenocarcinoma/high-grade dysplasia lesions, so it should be used along with standard four-quadrant biopsy protocol rather than alone (40).

A new generation AFI (AFI-III) is hypothesized to enhance early neoplasia detection from inflammation in Barrett's esophagus by specifically targeting fluorescence in malignant cells, thus reducing the false-positive rate. Boerwinkel et al. conducted an uncontrolled feasibility study of 45 patients with Barrett's esophagus to investigate the AFI-III system to detect early neoplasia. Out of 19 patients detected with high-grade intraepithelial neoplasia (HGIN)/early cancer, 47% (9/19) patients had lesions detected with white light endoscopy only, which was further improved to 79% (15/19) by AFE-II, then to 95% (18/19) by AFI-III and one final patient had lesion detected by random biopsies. The false-positive rate was 86% for both AFI-III and AFI-II, so this pilot study shows that AFI improves neoplasia detection in Barrett's esophagus but no additional benefit of AFI-III over AFI-II (38).

### Confocal Laser Endomicroscopy

Confocal Laser Endomicroscopy (CLE) is a technology developed for cellular and subcellular imaging up to 250 micrometers below the mucosal surface and thus provide real-time histology (*in-vivo*) during the procedure (36, 41). Confocal Laser Endomicroscopy combines a confocal laser microscope as a probe that can pass through the channel of an endoscope or as a tip of a standard video endoscope. White-light microscopy and confocal microscopy can be used simultaneously with confocal endoscopy technology, and a working channel can be utilized for target biopsies (26). In a clinical trial, 63 patients [long-lasting reflux symptoms (*n* = 20), Barrett's esophagus surveillance (*n* = 30), and suspected Barrett's -associated neoplasia (*n* = 13)] underwent CLE for *in vivo* diagnosis of Barrett's esophagus and associated neoplasia. This study showed that CLE could predict intestinal metaplasia and Barrett's esophagus-associated neoplasia with a sensitivity of 90.1 and 92.9%, a specificity of 94.1 and 98.4%, and accuracy of 96.8 and 97.4%, respectively. For the prediction of histopathologic diagnosis based on the confocal Barrett classification system, the mean kappa value for the interobserver agreement was 0.843, and for the intraobserver agreement was 0.892 (42).

A meta-analysis of 14 studies with 789 patients was performed to assess the accuracy of CLE for the diagnosis of high-grade dysplasia and esophageal neoplasia in Barrett's esophagus. Seven studies were included in the per-patient analysis, and corresponding pooled sensitivity and specificity were 89% (95% CI: 0.82–0.94) and 83% (95% CI: 0.78–0.86), respectively. For per-lesion analysis, ten studies were included, and corresponding pooled sensitivity and specificity were 77% (95% CI: 0.73–0.81) and 89% (95% CI: 0.87–0.90). Confocal Laser Endomicroscopy is a non-invasive, *in vivo* method for predicting neoplasm in Barrett's esophagus so that it could be used for neoplasm surveillance in Barrett's esophagus patients (43).

### Wireless Esophageal Capsule Endoscopy

Esophageal capsule endoscopy (ECE) was approved in 2004 to evaluate esophagus in patients with GERD and suspected Barrett's esophagus by Food and Drug Administration (FDA). It uses a video capsule endoscope, which has a camera at both ends. These cameras take pictures of the esophagus at 18 frames/s (44). A prospective multicenter trial of 89 patients with chronic reflux symptoms referred to five endoscopic centers for EGD was conducted to compare the diagnostic yield of ECE and EGD. Patients first underwent ECE and then EGD. Endoscopists who performed EGD were blinded to ECE, which was read by two independent readers. Out of 77 patients who completed the study, esophagitis, and endoscopically suspected esophageal metaplasia (ESEM) was present in 24 and 10 patients. The sensitivity, specificity, PPV, and NPV of ECE to detect esophagitis were 79, 94, 83, and 92%, respectively. The sensitivity, specificity, PPV, and NPV of ECE to detect ESEM and Barrett's esophagus were 60 and 71%, 100 and 99%, 100 and 83%, and 95 and 98%, respectively. For screening, ECE showed great specificity for esophagitis, ESEM, and Barrett's esophagus. However, it has a lower sensitivity for ESEM and Barrett's esophagus (45). A meta-analysis of nine studies with 618 patients showed pooled sensitivity and specificity of ECE to diagnose Barrett's esophagus of 77 and 86%, respectively. The pooled sensitivity and specificity of ECE for diagnosis of Barrett's esophagus using EGD as a reference and histologically confirmed intestinal metaplasia as reference were 78 and 78%, 90, and 73%, respectively (46).

## Advances in Endoscopic Treatments for Gerd

Interventional therapies for GERD and its complications can be divided into either surgical or endoscopic. Endoscopic therapies are a minimally invasive treatment option for patients who do not respond to medical therapy and do not want surgical intervention. Endoscopic therapies include radiofrequency ablation to lower esophageal, endoluminal suturing/plication, injection or implementation of biopolymers, endoscopic mucosal resection, endoscopic opposition devices as described below.

### Injectable Agents

#### Enteryx®

Enteryx® (Boston Scientific, Natick, MA) is a biocompatible polymer consisting of 8% ethylene vinyl alcohol mixed with radiopaque contrast agent (tantalum powder) in a solution of dimethyl sulfoxide, organic liquid carrier (19, 47). Enteryx® is liquid before injection, and it is injected within 1–3 mm of the esophagogastric junction in a circumferential pattern under fluoroscopic guidance. It turns into spongy mass after injecting into tissue, provides volume to the lower esophageal sphincter and reduces reflux (18, 19, 47) (Table 2).

**Table 2:** Different injectable agents used for endoscopic anti- reflux treatment.

**Injection agent name**	**Composition**	**FDA status**
Enteryx®	8% ethylene-vinyl alcohol copolymer mixed with tantalum dissolved in dimethyl sulfoxide.	Recalled from Market by manufacturers in 2015 due to complications including death.
Durasphere®	Carbon-coated beads containing zirconium oxide, suspended in a water-based, absorbable polysaccharide carrier gel.	Not approved by FDA for GERD treatment
Gatekeeper™	Soft pliable cushion polyacrylonitrile-based hydrogel prosthesis.	Removed from market due to poor long-term results
Plexiglas	Polymethylmethacrylate (PMMA)beads	Not approved by FDA for GERD treatment

*GERD, Gastroesophageal reflux disease; FDA, Food and Drug Administration*.

In an international multicenter clinical trial, 144 PPI-dependent patients with GERD were followed after Enteryx® implantation. PPI usage was reduced by more than 50 in 84% (95% CI: 76, 90%) and 72% (95% CI: 59, 82%) at 12 and 24 months, respectively. Similarly, PPI usage was eliminated in 73% (95% CI: 64, 81%) and 67% (95% CI: 54, 78%) at 12 and 24 months, respectively. Most adverse events occurred during the first 6 months, which resolved without long-term sequelae (48) (Table 3). In another multicenter trial, 64 patients with GERD on PPI were assigned to the Enteryx® implantation (*n* = 32) group and sham procedure consisting of standard EGD (*n* = 32) group. On 3 months follow-up, ≥50% reduction in PPI usage was higher in Enteryx®-treated patients (81%) than in the sham group (53%), with a rate ratio of 1.52 (95% CI: 1.06–2.28; *P* = 0.023). Similarly, PPI usage was eliminated in 68% of patients in the Enteryx® group vs. 41% in the sham group, with a rate ratio of 1.67 (95% CI: 1.03–2.80; *P* = 0.033). GERD health-related quality of life heartburn score improvement more than or equal to 50% was much high in Enteryx® group (67%) than sham group (22%) with a rate ratio of 3.05 (95% CI: 1.55–6.33; *p* < 0.001) (49). Although Enteryx® decreased PPI use and improved GERD score, it caused serious adverse events like embolization into vascular structures, transluminal injections, and even death leading to recall of this device in 2005 by the FDA (50–52).

**Table 3:** Studies with different injectable agents for endoscopic anti- reflux treatment.

**References**	**Device/injection agent**	**Number of patients (*n*)**	**Off PPI therapy after treatment**	**Common adverse events**
Cohen et al. (48)	Enteryx®	144	73% at 12 months and 67% at 12 months	Retrosternal chest pain, dysphagia. No serious adverse events.
Ganz et al. (53)	Durasphere®	10	NA at 6 months and 70% at 12 months	Pain around injection site, sore throat, nausea, bloating, chest pain, belching. No serious adverse events.
Fockens et al. (54)	Gatekeeper™	67	53% at 6 months and NA at 12 months	Sore throat, retrosternal or epigastric pain, nausea, vomiting, erosive duodenitis. No serious adverse events.
Feretis et al. (55)	Plexiglas	10	70% at 7.2 months (5–11 months)	Transient dysphagia, self-limiting bleeding. No serious adverse event

#### Durasphere®

Durasphere® (Carbon Medical Technologies, St Paul, Minnesota) is a bulking agent approved by the FDA in 1999 to treat urinary incontinence caused by bladder sphincter dysfunction. It is composed of carbon-coated graphite beads containing zirconium oxide, ranging from 90 to 212 mm, suspended in the water-based gel (2, 18, 19) (Table 2). A human pilot study of 10 patients with GERD on daily PPIs had an endoscopic injection at the gastroesophageal junction with Durasphere®. At 12 months follow-up, 90% of patients had >50% reduction in their PPI use, and 70% of patients discontinued all antacid medications. Four patients achieved normal pH scores, and the mean DeMeester scores improved from 44.5 to 26.2 at 12 months from baseline. Patients tolerated the procedure well with minor discomfort without adverse events (53) (Table 3). This study showed good results; however, it was a small sample and non-randomized study. Further large, randomized trials are needed. This device is not approved by FDA for GERD treatment.

#### Gatekeeper™

Gatekeeper™ reflux repair system (Medtronic, Minneapolis, MN) is another gastroesophageal bulking agent that restricts the distal esophagus's diameter by implanting a polyacrylonitrile-based hydrogel prosthesis into the submucosa of the cardia and lower esophageal junction (2, 19) (Table 2). In a study with pooled data from two prospective, non-randomized multicenter trials, 68 patients with GERD were treated with up to six Gatekeeper™ prostheses placed at the gastroesophageal junction. At 6 months, 24-h pH outcomes with pH < 4 for >4% of the time improved from 9.1 to 6.1% (*p* < 0.05). Patients who were no longer receiving PPI therapy reported significant improvement in median GERD heartburn-related quality-of-life score from 24.0 to 5.0 (*p* < 0.01). Serious events were reported in two patients, and both recovered uneventfully (54) (Table 3). A prospective multicenter randomized sham-controlled trial was started for this device, terminated early before completion due to infrequent severe adverse events. This device is no longer available in the market due to a lack of long-term data (2, 19).

#### Plexiglas

Another injectable agent is Plexiglas, an injection of polymethylmethacrylate (PMMA) beads, a highly viscous agent. The FDA has not approved it for endoluminal GERD treatment. However, it is approved as a biologically inert filler for cosmetic treatments (2) (Table 2). Feretis et al. conducted an only human study of endoscopic submucosal injection of Plexiglas in 10 patients with GERD who were either dependent or refractory to PPIs. After a follow-up of the mean of seven months, a significant decrease in symptoms severity and mean total time spent with esophageal pH < 4 was noted (*p* < 0.05). Seven of ten patients discontinued medication after the Plexiglas procedure (55) (Table 3). Although this study showed positive results, it is a small study with no long-term follow-up. No further human studies are available.

### Electrical Stimulation of the LES

The lower esophageal sphincter (LES) Electrical Stimulation with EndoStim® stimulation system (EndoStim BV, The Hague, The Netherlands) aims to augment the natural functioning LES by increasing LES pressure without affecting LES relaxation or peristalsis (2, 56). It obtained the CE mark in 2012. Currently, most of the studies involve the placement of this device laparoscopically (56, 57). Banerjee et al. conducted a study with a device placed endoscopically. In this study, a temporary pacemaker lead was placed endoscopically in the LES *via* a 3-cm submucosal tunnel in six patients with GERD. One patient had pre-mature lead dislodgement, and the remaining five had electric stimulation delivered 6–12 h post-implant per protocol. All patients had an increase in LES pressure after the procedure (58) (Table 4). There is also a recent porcine study using battery-device for electrical stimulation but no human studies available yet (59). Given that most human studies are available from laparoscopic studies, further large human studies with endoscopic implantation of devices are needed.

**Table 4:** Endoscopic procedure for GERD treatment.

**Procedure name**	**Evidence**	**Number of patients**	**Quality of life index (GERD-HRQL or GIQLI or SF-20)**	**Reduction/discontinuation of PPI use during follow up**	**DeMeester scores-measuring abnormal esophageal acid exposure**
Electrical stimulation of LES	Single center, feasibility study (58)	6 patients	N/A	N/A	N/A
Anti-reflux mucosectomy	Retrospective study (62)	109 patients	N/A	40–50% patients discontinued PPI on 6–12 months follow up	Improved to 24.9 ± 36.0, *p* < 0.01 at 2 months follow up
Radiofrequency ablation (Stretta)	Systematic review and meta-analysis of 28 studies (68)	2,468 patients	GERD-HRQL score improved by mean (random effects model) of −14.6 (−16.48, −12.73), *p* < 0.001	51% patients discontinued PPI	DeMeester score-pooled estimate (random effects model) of −13.79 (−20.01, −7.58), *p* < 0.001
Transoral incisionless fundoplication	Systematic review and meta-analysis of 32 studies (76)	1,475 patients	GERD-HRQL -Improved significantly to mean difference of 17.72 (95% Cl: 17.31–18.14), *p* < 0.001	89% patients discontinued PPI	Improved significantly by mean difference of 10.22 (95% Cl: 8.38–12.12, *p* < 0.0001)
Medigus ultrasonic surgical endostapler	Multicenter prospective trial (77)	66 patients	GERD-HRQL-improved significantly to mean (SD) −9.0 (9.1) on 6 months follow up	64.6% patients discontinued PPI on 6 months follow up	N/A
Endoscopic full-thickness plication (GERDx™)	Prospective study (79)	40 patients	GIQLI- Improved significantly to mean ± SD of 112.03 ± 13.11 (*p* < 0.001) at 3 months follow up	63.3% patients discontinued PPI on 3 months follow up	Improved to mean ± SD −20.03 ± 23.62 (*p* < 0.001) at 3 months follow up
Wilson-Cook endoscopic suturing device	Single center prospective study (83)	20 patients	GERD-HRQL. 50% patient reported improved in score but not statistically significant	Only 10% patients had reduction in PPI use at 6 months	DeMeester score improved to 47.1 (260.0–89.6), *p* = 0.54 at 6 months
BARD EndoCinch™	Single-center, double-blind, randomized, sham-controlled trial (96)	60 patients	Showed improvement in SF-20 at 6 and 12 months	≥50 and ≥95% reductions in 68 and 29% of patients at 12 months.	NA
NDO plicator	Multicenter, randomized, patient-blinded, sham-controlled trial (93)	159 patients	Showed significant improvement to mean ± standard deviation of 12.5 ± 11.1, *p* < 0.001 at 3 months	57% complete PPI cessation at 3 months, *p* = 0.001	Improved to [median (1st – 18 and 3rd -quartile)] −28 (18, 42) *p* = 0.001 at 3 months
Anti-reflux device	Multicenter study (94)	70 patients	Mean GERD-HRQL improved to 69% at 6 months follow up	63% patients off anti-secretory medications at 6 months.	N/A
His-Wiz anti-reflux procedure	Prospective pilot study (95)	7 patients	N/A	57.14% patients off anti-secretory medications	N/A
Endoscopic band ligation	Single center prospective study (80)	150 patients	GERD-HRQL score improved to mean ± SD of 14.7 ± 3.9 at 1 year follow up	N/A	N/A
Peroral endoscopic cardial constriction	Preliminary follow up study (81)	13 patients	GERD-HRQL score improved to mean ± SD of 4.46 ± 4.31 and 5.69 ± 5.07 at 3 and 6 months follow up	N/A	Improved to mean ± SD of 16.97 ± 12.76 and 20.32 ± 15.22 at 3 and 6 months follow up
Resection and plication	Prospective study (82)	10 patients	GERD-HRQL Score showed absolute reduction 22.3, (95 % CI 19.3 – 25.3), *p* < 0.0001 on median 9 months follow up	80% stopped using PPI on median 9 months follow up	N/A

*GERD-HRQL, Gastroesophageal disease health-related quality of life; SF-20-, 20-item Short-Form Health Survey; SD, Standard Deviation; PPI, proton pump inhibitors*.

### Anti-reflux Mucosectomy

Anti-reflux mucosectomy (ARMS) is a technique that involves hemi-circumferential mucosal resection of gastric cardia around the esophagogastric junction. The mucosal healing leads to scar formation, which in turn results in narrowing of the gastric cardia opening and thus reducing reflux episodes (2, 4, 18, 60). This technique is derived from circumferential mucosal resection for Barrett's esophagus with short segment high-grade dysplasia as these patients reported significant improvement in their GERD symptoms after mucosal resection (2). This procedure was first described in a pilot study where ten patients with treatment-refractory GERD underwent the ARMS procedure. Patients reported significant improvement in GERD symptoms. In the DeMeester score, the mean heartburn score improved from 2.7 to 0.3 (*p* = 0.0011), regurgitation score improved from 2.5 to 0.3 (*p* = 0.0022) (61). In a retrospective study of 109 patients with PPI-refractory GERD, 40–50% of patients were able to discontinue PPIs after ARMS. The Acid Exposure Time and DeMeester Score improved significantly from 20.8 ± 24.3 to 6.9 ± 10.4 (*p* < 0.01) and 64.4 ± 75.7 to 24.9 ± 36.0 (*p* < 0.01), respectively, at the end of 2 months. However, there was no significant improvement in the number of proximal reflux episodes (*p* = 0.0846). After 2–3 weeks, transient stenosis was reported in 13 patients requiring balloon dilation (62) (Table 4). Although this procedure is shown to be effective in studies, there are no large long-term randomized trials available. So, a randomized trial showing long-term benefits is needed before recommending it widely.

### Radiofrequency Ablation (Stretta)

The Stretta system (Mederi Therapeutics, Norwalk, CT, USA) is a radiofrequency energy application to the distal esophagus, GEJ, and cardia of the stomach. In this endoscopic procedure, thermal energy is delivered at a temperature range of 65–85° to the muscle of the lower esophageal sphincter and gastric cardia *via* a 4-channel radiofrequency generator and catheter system equipped with four needle electrodes. The exact mechanism of action is not clear, but the proposed mechanism includes hypertrophy of muscularis propria after the procedure and decreases transient LES relaxation (2, 4, 63) (Supplementary Figures S3, S4). It was approved by the FDA in 2000 and recommended by the Society of American Gastrointestinal and Endoscopic Surgeons (SAGE) (64, 65). In an RCT, 64 patients with GERD were randomized to either radiofrequency energy delivery group (active treatment, *n* = 35) or a sham procedure (*n* = 29). More than 50% improvement in GERD HRQL score was seen in the active treatment group than sham procedure group (61 vs. 30%, *p* = 0.03), and similarly, more patients in the active treatment group were without daily heartburn symptoms than sham group (61 vs. 33%, *p* = 0.05). There was no statistically significant difference in acid-suppressive medication use and esophageal acid exposure between the two groups at 6 months follow-up (66).

In a meta-analysis of four RCTs with 165 patients, pooled results did not show any difference in sham or Stretta procedure or management with PPI in patients with GERD for esophageal acid exposure, lower esophageal sphincter pressure, ability to stop PPIs or GERD-HRQL outcomes. However, the overall quality of evidence was low (67). In another meta-analysis of 28 studies (four RCTs, 23 cohort studies, and one registry) with 2,468 patients, pooled results showed a significant improvement in GERD HRQL score and heartburn standardization score by −14.6 and −1.53, respectively. Stretta treatment also led to statistically significant improvement in esophageal acid exposure time and incidence of erosive esophagitis (*p* < 0.001) (68) (Table 4). Stretta is an outpatient procedure that can be performed under conscious sedation. It is shown to be safe and effective in most studies (66, 67, 69–71).

### Transoral Incisionless Fundoplication

Transoral incisionless fundoplication (TIF), with the use of the EsophyX® device, is a minimally invasive treatment of GERD, which was introduced as an endoscopic substitute for surgical reconstruction of the LES. This procedure endoscopically reconstructs the LES to restore the angle to His (the acute angle between the cardia and the esophagus) (2, 72). TIF was initially introduced as endoluminal fundoplication in 2005 and then underwent several modifications in 2007 (TIF 1.0) and 2009 (TIF 2.0). In TIF 1.0, fasteners were placed 1 cm above the GEJ junction, and no circumferential wrap was created, whereas, in TIF 2.0, fasteners were placed 1–3 cm above the GEJ junction using a retroflexed flexible endoscope and create a 270-degree wrap using EsophyX® device (60, 72) (Supplementary Figure S5). The FDA cleared the EsophyX® device in September 2007 (72).

The RESPECT (Randomized EsophyX2 vs. Sham, Placebo-Controlled Transoral Fundoplication) study was a multicenter RCT comparing the TIF procedure plus 6 months of placebo medication (*n* = 87) vs. a sham operation and optimal PPI therapy for 6 months (control, *n* = 42) for patients with troublesome regurgitation despite daily PPI use. By intention-to-treat analysis, a higher proportion of patients with TIF reported eliminating troublesome regurgitation than the control group (67 vs. 45%, *p* = 0.023). GERD symptoms score improved in both groups, but control of esophageal pH improved after TIF only (mean 9.3% before and 6.3% after, *p* < 0.001), not sham surgery (mean 8.6% before and 8.9% after) (73).

The TIF 2.0 EsophyX® vs. Medical PPI Open-label (TEMPO) trial randomized multicenter trial compared the efficacy of TIF (*n* = 40) and high dose PPIs (*n* = 23) in patients with troublesome regurgitation and extraesophageal symptoms of GERD. Troublesome regurgitation eliminated in 97% of TIF vs. 50% of PPI patients [Risk Ratio (RR) = 1.9; 95% Cl = 1.2–3.11; *p* = 0.006] at 6-month follow up. GERD health-related quality of life (GERD-HRQL) score improved significantly in the TIF group (from 19 to 2, *p* < 0.001) compared to lesser improvement in the PPI group (from 17 to 11, *p* = 0.012) at 6 months (74). On long-term follow-up, troublesome regurgitation and atypical symptoms resolution was achieved in 86 and 80% of patients, respectively, at 5 years. The total GERD-HRQL score improved to 6.8 from 22.2, *p* < 0.001 at 5 years. No serious adverse events were reported during this follow-up period (75). A meta-analysis and systematic review of 32 studies with 1,475 patients showed TIF success rate was 99% (95% Cl: 97–100; *p* < 0.001) and an adverse event rate of 2% (95% Cl: 1–3; *p* < 0.001). After TIF procedure, GERD-HRQL, DeMeester Score, and Reflux Symptom Index (RSI) improved significantly (mean difference 17.72, 95% CI: 17.31–18.14; mean difference 10.22, 95% CI: 8.38–12.12; mean difference 14.28, 95% CI: 13.56–15.01; *p* < 0.001). PPIs was discontinued in 89% of patients (95% Cl: 82–95; *p* < 0.001) (76) (Table 4). TIF is a safe, viable, and promising endoscopic option for patients with refractory GERD symptoms.

### Medigus Ultrasonic Surgical Endostapler

The Medigus Ultrasonic Surgical Endostapler (MUSE™) (Medigus, Omer, Israel) is an endoscopic stapling device for transoral partial fundoplication (4). The complete device consists of a flexible endoscope, an endo stapler, a miniature video camera, and an ultrasound transducer. The MUSE™ endoscope is advanced into the stomach through a previously placed overtube, retroflexed, and then the device is pulled back until the chosen stapling level (usually 3 cm above GEJ). Subsequently, a staple is delivered under the guidance of an ultrasound gap finder, and the process is repeated to form a 180-degree fundoplication (2, 4, 60). This device was first cleared in January 2015 by the FDA (2).

In a multicenter prospective clinical study, 66 patients were followed for 6 months after endoscopic fundoplication using MUSE™ for GERD. At 6 months follow up, more than 50% decrease in GERD-HRQL score was achieved in 73% (95% Cl: 60–83%), and 64.6% of patients stopped taking PPIs or any other acid reduction medications. Eight adverse events occurred in the first 24 subjects, including pneumomediastinum, pneumoperitoneum, pleural effusion, upper gastrointestinal bleeding, and esophageal leak. After an interim review of these early adverse events, protocol and device changes were implemented, leading to reduced adverse events, and no other cases of leak or pneumomediastinum were reported (77) (Table 4). In a study evaluating long-term results of endoscopic treatment of GERD with MUSE™ device, 83.8% at 6 months and 69.4% of patients at 4 years remained off PPIs. GERD-HRQL score of the total patients improved from 29.1 ± 5.6 to 5.3 ± 5.8 (*p* < 0.01) at 4 years after the procedure. The daily dosage of GERD medications, measured as omeprazole equivalents, improved from 66.1 (±33.2) to 10.8 (±15.9) and 12.8 (±19.4) at 6 months and 4 years, respectively (*p* < 0.01) (78). Although MUSE™ is effective, limited data is available, so further randomized trials with long-term outcomes are needed.

### Endoscopic Full-Thickness Plication (GERDx™)

Endoscopic full-thickness plication was initially carried out using a plicator device (Ethicon Endosurgery, Somerville, NJ, USA), which is no longer available. A new device, the GERDx™ system (G-SURG GmbH, Seeon-Seebruck, Germany), was produced and introduced by a different manufacturer. The procedure involves endoscopic full thickness gastroplication using this device and a flexible endoscope (2, 4, 18). In a prospective study, 40 patients with GERD underwent endoscopic plication with GERDx™ device. Seven of forty patients underwent laparoscopic fundoplication before 3 months follow-up, and three additional patients did not want to further participate in the study, so 30 patients were available at the 3-month follow-up. The mean DeMeester score improved from 46.48 (±30.83) to 20.03 (±23.62) at 3 months (*p* < 0.001). The mean gastrointestinal quality of life index (GIQLI) improved from 92.45 (±18.47) to 112.03 (± 13.11) at 3 months. Sore throat (20%) and chest pain (17.5%) were the most common reported adverse events and whereas four patients had serious adverse events, including hematoma at the gastroesophageal junction, Mallory Weiss lesion, pneumonia with pleural effusion, intractable post-operative pain requiring laparoscopic suture removal (79) (Table 4). There is currently limited data regarding GERDx™, so further randomized controlled trials are needed before implementing it in routine clinical practice.

### Emerging Gastroesophageal Junction-Altering Techniques

Three additional emerging GEJ altering techniques have been described that utilize endoscopic band ligation or peroral endoscopic cardiac constriction or resection and plication (RAP) to reduce gastric cardia opening. In an RCT of 150 patients with refractory GERD, 75 patients were assigned to the endoscopic banding ligation group (banding done at four quadrants just at GEJ) and the other 75 to the control group (optimized dose of PPIs). These patients were followed for 1 year and reported significant improvement in GERD-HRQL, the site of the Z line, with signification reduction in reflux episodes when compared to the medical treatment group. No major adverse events were reported; mild dysphagia and epigastric pain were the only reported adverse events (80) (Table 4).

Hu et al. described a new technique, peroral endoscopic cardial constriction for gastric cardiac constriction. In this procedure, two single-band ligation devices were placed at greater and lesser curvature under endoscopic guidance, and subsequently, the two ends of ligation devices were fixed with resolution clips. A total of 13 patients underwent the procedure successfully. At 3 and 6 months follow up, the GERD-HRQL scale was 4.46 (±4.31) and 5.69 (±5.07), respectively, from a baseline of 19.92 (±7.89). Similarly, at 3 and 6 months follow up, DeMeester score improved to 16.97 (±12.76) and 20.32 (± 15.22), respectively, from a baseline of 125.50 (± 89.64). There were no serious complications; slight retrosternal pain and dysphagia were reported in 3 patients. This study shows that peroral endoscopic cardial constriction is a safe and effective method for the treatment of GERD. However, it is a small preliminary clinic study, so further data is needed (81) (Table 4).

Benias et al. described a novel resection and plication (RAP) procedure, limited crescent-shaped mucosectomy at the level of the gastroesophageal junction followed by full-thickness plication of the LES using Apollo Overstitch (Apollo Endosurgery, Austin, Texas) in a pre-determined pattern. In this pilot study, 10 patients with GERD symptoms refractory to PPI underwent the RAP procedure. All patients were discharged the same day from the hospital after the procedure without any adverse events. During mean 9 months (range 5–24 months) follow-up, all patients had significant improvement in GERD-HRQL scores, and daily PPI dependence was eliminated in 8 out of 10 patients (82) (Table 4).

These techniques have only limited data available. Further randomized studies comparing these techniques with other current standards of care are needed.

### Wilson-Cook Endoscopic Suturing Device

The Endoscopic Suturing Device (ESD) (Wilson-Cook Medical Inc., Winston-Salem, NC) is a single-use endoscopically assisted endoluminal suturing device, which was first introduced in 2002 (83). It has three components- an external accessory channel, a flexible Sew-Right device, and a flexible T-Knot device (84). Both Sew-Right and T-Knot devices are inserted through an external accessory channel attached to a flexible endoscope, and the true working endoscope channel of the endoscope can be used for further interventions as needed (83, 84). A single-center prospective study of 20 patients with GERD who failed treatment with EndoCinch underwent an ESD procedure. Technical success was 100%, but no significant changes in the 24-h pH monitoring results based on a median pH < 4/24 h after treatment when compared with baseline (9.9 vs. 12.3%, *p* = 0.60) were seen after 6 months. Similarly, there was no significant change in the PPI use and manometry finding (median LES pressure 7.2 vs. 9.9 mmHg, *p* = 0.22). Only 5% of patients were found to have sutures *in situ* at 6 months follow up (83) (Table 4). A clinical phase of another uncontrolled study of 20 patients with GERD also showed poor clinical outcomes. There was no significant improvement in PPI use, LES pressure on manometry, pH study. Only 12% of plication persisted at 3 months follow up (84). Both studies showed early suture loss. The ESD is no longer available or market for clinical use (19).

### BARD EndoCinch™

The BARD EndoCinch ™ (C.R. Bard Inc., Murray Hill, NJ, USA) is used for endoluminal gastroplication (85). This procedure was first described by Swain and Mills in 1986 and approved by the FDA in 2000 (86). The EndoCinch procedure uses a sewing capsule attached to the distal tip of an endoscope to create partial-thickness pleats through a series of sutures at the gastric cardia (87). In a multicenter prospective, open-labeled trial, 48 patients with GERD underwent endoluminal gastroplication using the EndoCinch™ system. For 24 months follow-up period, the rate of complete resolution of heartburn symptoms ranged from 54 to 66%, the rate of successful discontinuation of PPI or H2 receptor antagonist ranged from 65 to 76%. The rate of patients who had successful discontinuation of PPI or H2 receptor antagonist, improvement in endoscopic Los Angeles classification to grade O, improvement in heartburn symptoms were greater in patients with more than one plication remaining than with loss of all plications (88). A study evaluating long-term effects of EndoCinch™ treatment showed that in the 4-year follow-up period, 44% of patients needed retreatment after a median period of 4 months (interquartile range 3–8), and 80% required PPI again for their GERD symptoms (89). EndoCinch ™ fails to show long-term benefits for most patients with GERD (89, 90). Furthermore, it is shown to be inferior to surgical fundoplication (91).

### NDO Plicator

The NDO is a full thickness suturing transmural plicator designed by NDO Surgical Inc. (Mansfield, MA) in 2003, and the FDA cleared the device in May 2004 (86, 87). This device uses a pretied suture-based implant to secure a plication near the gastroesophageal junction under the visualization of a flexible endoscope. It creates a transmural full-thickness plication with serosa-to-serosa fusion at the angle of His (19, 87). In 2003, a pilot study of the use of endoscopic full-thickness plication in patients with chronic heartburn and pathologic reflux showed a reduction in heartburn score, anti-GERD medication use. Only mild adverse events were reported, which resolved spontaneously within 7 days of the procedure (92). In a prospective RCT, patients were randomly assigned to the active group, endoscopic full-thickness plication (*n* = 78), and sham group (*n* = 81). By intent-to-treat analysis, patients achieving ≥50% improvement in GERD-HRQL score were significantly higher in the active group (56%) than the sham group (18.5%) at 3 months (*p* < 0.001). Similarly, it shows a higher PPI cessation in the active group than the sham group (50 vs. 24%, *p* = 0.002). No perforation or deaths were reported (93) (Table 4). This device is no longer available for commercial use as it was taken off the market in June 2008 due to the company's poor financial performance (19, 86).

### Anti-Reflux Device

Anti-Reflux Device (Syntheon, Miami, FL, USA) is a titanium compression implant that creates a full-thickness plication in the gastric cardia along the anteriorly contiguous to the lesser curve to create a serosa-to-serosa apposition (19, 94). It allows using a standard gastroscope without overtube as the device can be passed alongside the gastroscope and controlled independently. The gastric wall is pulled into the Anti-Reflux device's jaws using a catheter-based tissue retractor through an endoscope biopsy channel, and then a titanium implant is deployed as jaws close to creating a full-thickness pleat. In a multicenter trial, 70 patients with symptomatic chronic GERD dependent on daily anti-secretory medications were treated with Anti-Reflux Device. At 6 months of follow-up, 79% of patients had ≥50% improvement in GERD-HRQL scores, and 63% were off anti-secretory therapy. The most common adverse event reported was epigastric/referred chest pain (31%), and one patient with prior history of complicated peritoneal infection had gastric perforation. The patient had an uneventful recovery after surgical intervention (94) (Table 4). Anti-reflux Device has not been brought forward for commercialization (19).

### The His-Wiz Anti-Reflux Procedure

The His-Wiz (Apollo Group/Olympus Optical, Tokyo, Japan) is a novel, overtube-based endoscopic device that allows for infrasphincteric application resulting in the accentuation of the gastroesophageal barrier. This device allows for full-thickness suturing and automatic cutting ability in a single-step procedure (19, 95). In a prospective pilot study, seven patients with chronic GERD on maintenance anti-secretory therapy underwent a 2-plication approach where two plications were performed on the anterior and posterior walls below the GEJ. Patients reported improvement in heartburn scores and pH monitoring, although a trend toward worsening anti-reflux was seen at 1 year. Most adverse events were transient and minor except for one patient with significant bleeding requiring endoscopic therapy. This was a small study (95) (Table 4). This device has not been brought forward for commercialization yet (19).

## Future Directions

There have been significant advancements in endoscopic diagnosis and treatment of GERD over the last two decades. Newer advanced endoscopic imaging technologies show promising results in improving diagnosis accuracy. Endoscopic therapies provide a minimally invasive option for patients who are not responding to medical therapies and for patients with prior fundoplication and bariatric surgeries. However, large randomized, long-term studies are needed to show the efficacy of these procedures compared to traditional surgical and laparoscopic procedures. Although these endoscopic therapies have shown improvement in quality of life and patient symptoms, they have not shown consistent results in objective parameters like augmentation of LE pressure, esophageal acid exposure, and pH normalization.

## Conclusion

Newer advanced endoscopic imaging and intervention techniques can improve the diagnostic accuracy of GERD and could improve target biopsy samples from high yield areas. This could decrease unnecessary biopsies from non-dysplastic areas, identifying abnormal mucosal or vascular patterns of lesions that could improve outcomes. However, these imaging techniques are still not very prevalent outside large academic institutions, likely due to limited access to training and the need for additional equipment. A growing number of patients fail to respond to pharmacological therapy with acid suppressant medications like PPI, and in these patients, endoscopic techniques for GERD are a minimally invasive option to surgical intervention. These endoscopic interventions are for the well-selected patient population. An endoscopic intervention like bulking injection agent and endoscopic suturing techniques showed varying degrees of response and did not show long-term efficacy. Techniques like radiofrequency treatment and endoscopic fundoplication are showing more promising results. These endoscopic techniques could be an alternative option for patients who are not good surgical candidates and have GERD refractory to PPI or GERD complications. Long-term randomized trials are needed comparing pharmacological, endoscopic, and surgical intervention for GERD treatment.

## Author Contributions

RM, MG, CU, AP, and HG: conception and design. RM, MG, and CU: literature search. RM and MG: first draft. All authors: critical revision, editing, and final approval.

## Conflict of Interest

The authors declare that the research was conducted in the absence of any commercial or financial relationships that could be construed as a potential conflict of interest.

## Publisher's Note

All claims expressed in this article are solely those of the authors and do not necessarily represent those of their affiliated organizations, or those of the publisher, the editors and the reviewers. Any product that may be evaluated in this article, or claim that may be made by its manufacturer, is not guaranteed or endorsed by the publisher.

## Supplementary Material

Supplementary Figure S1 Narrow band imaging endoscopic image showing vascular patter. Increased squamocolumnar junction vascularity (arrows). (Reproduced from Sharma et al. (97), with permission from Elsevier).

Supplementary Figure S2 Flexible spectral Imaging Color Enhancement (FICE) filter factor 0 image showing minimal esophagitis (Reproduced from Negreanu et al. (98), with Creative Commons Attribution Non-Commercial License).

Supplementary Figure S3 Radiofrequency Ablation (Stretta®) procedure- Stretta® catheter with inflated balloon (Reproduced from Utley et al. (100), with permission from Elsevier).

Supplementary Figure S4 Radiofrequency Ablation (Stretta®) procedure- showing antegrade and gastric cardia rings (Reproduced from Utley et al. (100), with permission from Elsevier).

Supplementary Figure S5 Transoral incisionless fundoplication (TIF), with the use of EsophyX® device **(A)** showing creation of esophagogastric fundoplication; **(B)** Showing post EsophyX® appearance (Reproduced from Jain et al. (99), with Creative Commons Attribution Non-Commercial License).
