# Supplementary figures and images for: Advanced Endoscopic Imaging and Interventions in GERD: An Update and Future Directions
Source: Front Med (Lausanne). 2021 Nov 29;8:728696. doi: 10.3389/fmed.2021.728696 (PMC8666712; doi:10.3389/fmed.2021.728696)

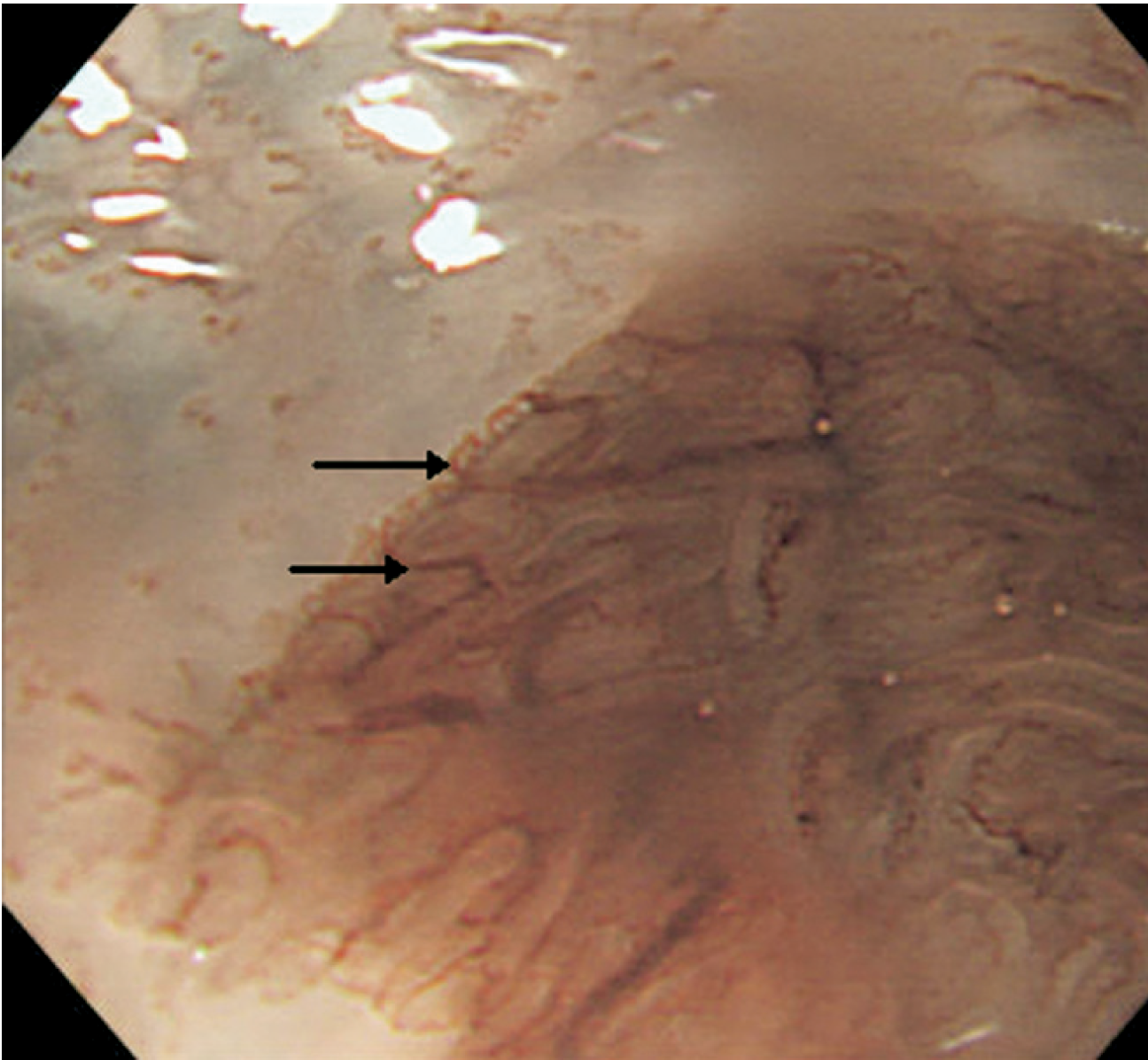

Supplement: Supplementary Figure S1 — Narrow band imaging endoscopic image showing vascular patter. Increased squamocolumnar junction vascularity (arrows). (Reproduced from Sharma et al. (97), with permission from Elsevier). [file Image_1.TIF]

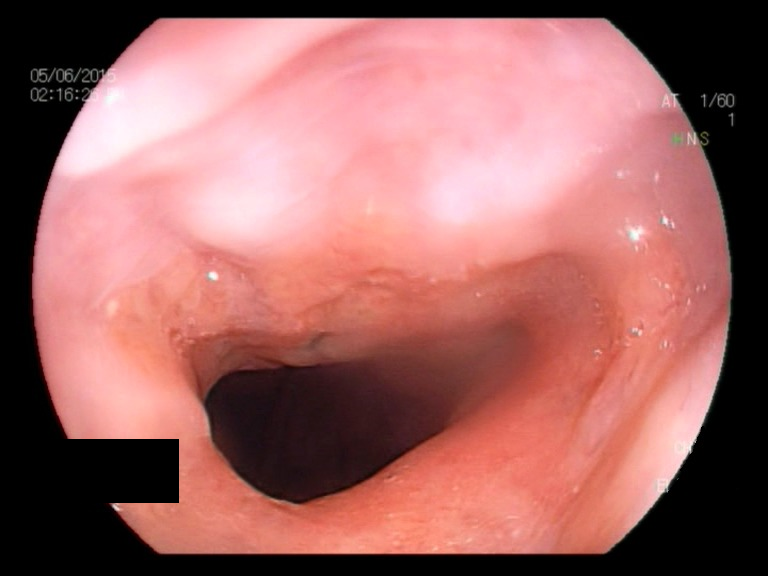

Supplement: Supplementary Figure S2 — Flexible spectral Imaging Color Enhancement (FICE) filter factor 0 image showing minimal esophagitis (Reproduced from Negreanu et al. (98), with Creative Commons Attribution Non-Commercial License). [file Image_2.TIF]

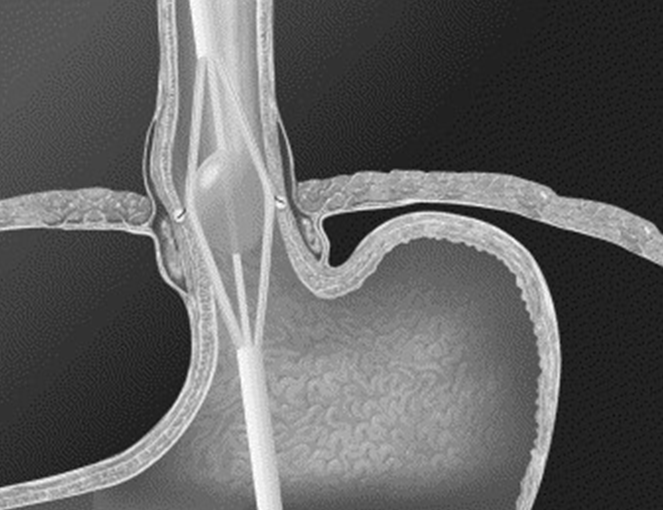

Supplement: Supplementary Figure S3 — Radiofrequency Ablation (Stretta®) procedure- Stretta® catheter with inflated balloon (Reproduced from Utley et al. (100), with permission from Elsevier). [file Image_3.TIF]

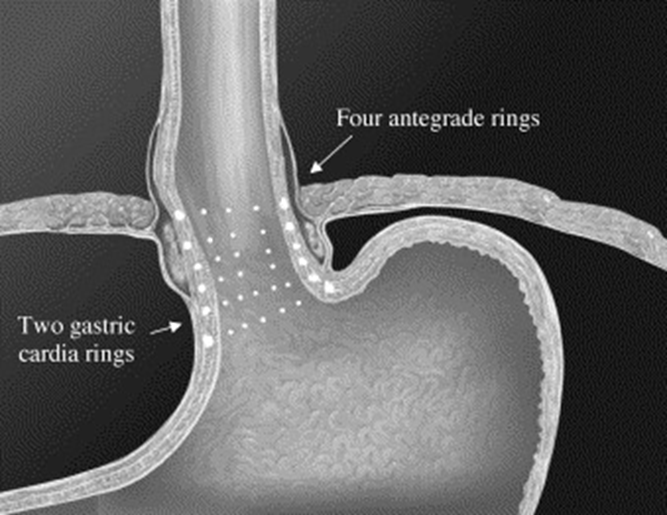

Supplement: Supplementary Figure S4 — Radiofrequency Ablation (Stretta®) procedure- showing antegrade and gastric cardia rings (Reproduced from Utley et al. (100), with permission from Elsevier). [file Image_4.TIF]

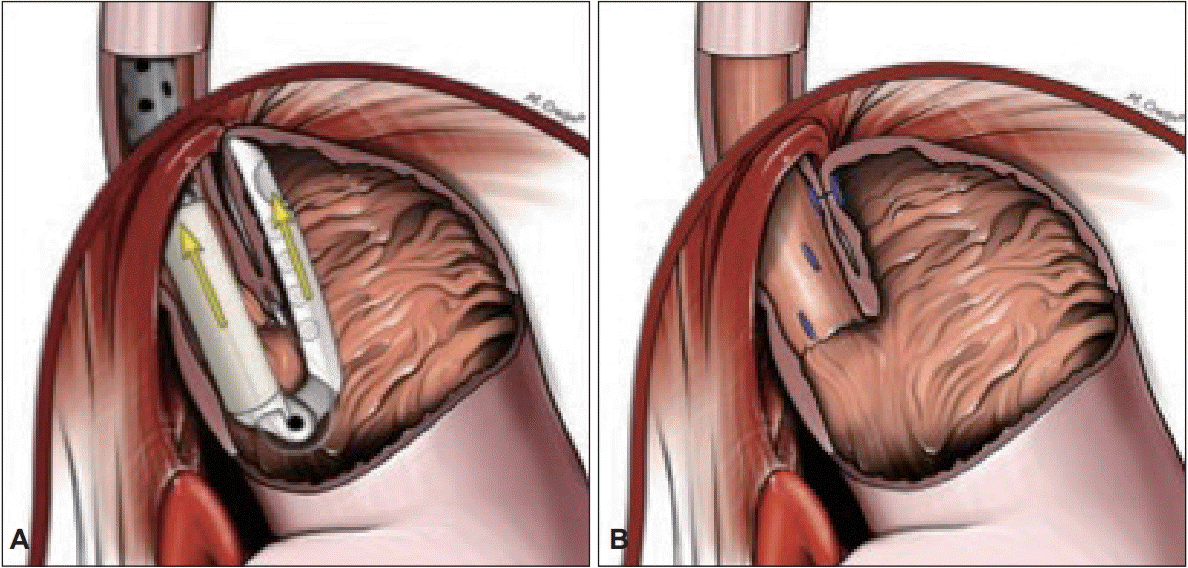

Supplement: Supplementary Figure S5 — Transoral incisionless fundoplication (TIF), with the use of EsophyX® device (A) showing creation of esophagogastric fundoplication; (B) Showing post EsophyX® appearance (Reproduced from Jain et al. (99), with Creative Commons Attribution Non-Commercial License). [file Image_5.TIF]
